# Recent Advances in the Detection of Food Contaminants and Pollutants

**DOI:** 10.3390/foods15061011

**Published:** 2026-03-12

**Authors:** Giulia Simonetti, Francesca Buiarelli

**Affiliations:** Department of Chemistry, “Sapienza” University of Rome, P.le Aldo Moro 5, 00185 Rome, Italy

Foods are essential sources of nutrients and energy for human populations; however, their quality and safety are continuously challenged by a wide range of factors acting along the food production chain. Physicochemical transformations occurring during processing, storage, and aging—such as oxidation, hydrolysis, and lipid degradation—can alter sensory attributes and nutritional value, while also affecting matrix composition and stability. In parallel, foods may contain intentionally added substances, non-intentionally added substances originating from food contact materials, and environmental pollutants introduced at different stages of production and distribution [1]. The coexistence of these processes and contaminants not only complicates food quality assessment but also raises significant concerns regarding consumer safety and human health.

In recent decades, the analytical control of contaminants in foods has therefore become increasingly complex. Target compounds span a broad spectrum of chemical classes, including inorganic elements, mycotoxins, plastic additives, veterinary drug residues, pesticides, and other emerging pollutants, often present at trace or ultra-trace levels in highly heterogeneous matrices. Their diverse physicochemical properties frequently preclude the use of a single universal analytical strategy, making multi-technique and integrated approaches essential. Within this framework, mass spectrometry (MS), particularly when coupled with separative techniques such as chromatographic or electrophoretic, coupled to tandem detection (MS/MS), has established itself as the reference technology for confirmatory analysis. Its high sensitivity, selectivity, and versatility enable reliable targeted and non-targeted workflows, supporting accurate identification and quantification even in complex food and food-related matrices [2,3,4,5].

Alongside laboratory-based platforms, increasing attention has been directed toward complementary methodologies aimed at enhancing screening capacity, reducing analytical turnaround times, and enabling earlier monitoring along the food production chain. Miniaturized spectroscopic instruments, advanced chemometric data processing, and sensor-based systems are increasingly being explored as tools for rapid, on-site assessment of food quality and safety [6,7,8,9]. Rather than replacing high-resolution mass spectrometry, these approaches contribute to a more integrated analytical framework, in which early detection and field-based screening operate synergistically with laboratory confirmation, thereby improving the overall efficiency and robustness of food safety control systems.

Building on this integrated analytical framework, this Special Issue was conceived to provide an overview of recent methodological developments in the field of food contaminant and pollutant analysis, with particular emphasis on mass spectrometry and its integration with alternative or complementary techniques for the comprehensive characterization of foods and food-related matrices.

The contributions include applications of portable spectroscopy for raw material screening, long-term monitoring of toxic metals in seafood, analytical approaches for emerging mycotoxins, sensor platforms for dairy monitoring, and LC-MS/MS methods for endocrine-disrupting compounds in packaging-related matrices [10,11,12,13,14,15].

In particular, the five contributions included in this Special Issue illustrate the practical implementation of these analytical strategies across different contaminants, matrices, and points along the food production chain. One [Contribution 1] study explores the application of portable near-infrared spectroscopy combined with chemometric modeling for rapid classification of brewing malts, highlighting how miniaturized analytical platforms can support quality control directly at industrial sites and reduce the risk of downstream non-compliance. Although not focused on a specific contaminant, this work exemplifies the growing relevance of fast screening tools in complex supply chains.

A second contribution [Contribution 2] addresses long-term monitoring of toxic metals in seafood products using inductively coupled plasma mass spectrometry and atomic absorption techniques. The results underline the continued relevance of elemental contaminants such as mercury, cadmium, and lead, particularly in marine ecosystems and high-trophic-level species, and confirm the central role of MS-based techniques in official control and exposure assessment.

The detection of emerging mycotoxins is examined through a comparative evaluation of capillary electrophoresis, liquid chromatography with diode-array detection, and LC-MS/MS for the determination of moniliformin in maize. This study [Contribution 3] illustrates how combining separation techniques with tandem mass spectrometry is often indispensable for analytes that are highly polar, weakly retained in conventional chromatographic systems, and insufficiently covered by current regulations.

Another contribution [Contribution 4] focuses on sensor platforms for milk monitoring, discussing methodological aspects of data acquisition, processing, and interpretation, as well as practical limitations encountered during field deployment. The study emphasizes how sensor-based technologies can function as early-warning systems, guiding the selective application of high-end analytical methods such as MS for confirmatory testing and supporting more efficient quality control strategies in the dairy sector.

Finally, the development and validation of an LC-MS/MS method for the determination of bisphenols in bottled water and PET/rPET packaging matrices addresses a highly topical issue related to food contact materials and non-intentionally added substances. The work [Contribution 5] highlights the analytical challenges associated with endocrine-disrupting compounds present at trace levels and demonstrates the necessity of sensitive MS-based approaches to support both risk assessment and regulatory compliance in the context of sustainable packaging.

Taken together, the collection of studies in this Special Issue confirms that the analytical control of food contaminants is increasingly defined by methodological integration rather than technological substitution. While high-resolution MS remains central for definitive identification and quantification, spectroscopic, electrophoretic, and sensor-based techniques expand the analytical toolbox, providing support for screening, monitoring, and process control across diverse matrices and stages of the food production chain.

Future progress in food safety will depend not only on further improvements in instrumental performance but also on the development of harmonized validation protocols, robust chemometric and data-analysis strategies, and closer integration between laboratory-based and in-field analytical systems. By presenting recent examples of these converging trends, this Special Issue aims to contribute to a broader understanding of how advanced analytical chemistry can support safer and more transparent food systems in an increasingly complex global context.

## Data Availability

Not applicable.
